# Transcription factor regulation and cytokine expression following in vitro infection of primary chicken cell culture with low pathogenic avian influenza virus

**DOI:** 10.1186/1743-422X-10-342

**Published:** 2013-11-19

**Authors:** Haijun Jiang, Kangzhen Yu, Darrell R Kapczynski

**Affiliations:** 1 Exotic and Emerging Avian Disease Research Unit, Southeast Poultry Research Laboratory, Agricultural Research Service, USDA, 934 College Station Road, Athens, GA 30605, Greece; 2 Key Laboratory of Zoonosis of Ministry of Agriculture, College of Veterinary Medicine and State Key Laboratory of Agrobiotechnology, China Agricultural University, Beijing, People‘s Republic of China

**Keywords:** Avian influenza virus, Chicken, Cytokine, Transcription factor

## Abstract

**Background:**

Avian influenza virus (AIV) induced proinflammatory cytokine expression is believed to contribute to the disease pathogenesis following infection of poultry. However, there is limited information on the avian immune response to infection with low pathogenic avian influenza virus (LPAIV).

**Methods:**

To gain a better understanding of the early viral-host interactions of LPAIV in chickens, primary chicken embryo hepatocytes (CEH) were infected with four different LPAIVs of U.S. origin. Kinetics of virus replication, transcription factor (c-Jun, p50 and IRF-3) activation and immune response gene (IL-6, IL-1beta, IFN-alpha and Mx) expression were studied at four different time points (6, 12, 24 and 48 hours) post infection and compared to non-infected controls.

**Results:**

CEH can support growth of the tested LPAIVs when with trypsin supplementation. All four immune response genes tested were upregulated following infection as were transcription factors c-Jun, p50 and IRF-3. Amplification of these genes was dependant on virus replication (e.g. inclusion of trypsin), such that immune response genes and transcription factors were upregulated as viral titers increased.

**Conclusion:**

The results of these studies demonstrate the requirement of virus replication for innate immune regulation and broaden our understanding of transcription factor responses related to LPAIV infection in chickens.

## Introduction

Avian influenza (AI) infection in poultry can result in high morbidity and mortality, and negatively affect international trade [1]. AIV can be categorized into 16 different hemagglutinin (HA) subtypes (H1–H16) and 9 different neuraminidases (N1–N9) subtypes which can occur in many combinations [2]. During the past decade, low pathogenic (LP) AIV has caused considerable economic loss due to decreased production, increased morbidity and increase the cost of vaccination in poultry industry [3]. LPAI viruses have the potential to evolve into highly pathogenic (HP) AIV and this has been documented in many poultry outbreaks. However, only the H5 and H7 subtypes, have been associated with HPAI mutation [4,5]. This has led to a new proposed definition of avian influenza to extend all infections caused by H5 and H7 viruses regardless of their virulence as notifiable avian influenza (NAI) [6]. The H9N2 subtype was first reported to infect turkeys in the United States in 1966 and has seriously affected the poultry industry of the Asia and Middle East since the mid-1990s [7-9]. This subtype is considered one of the most likely candidates to cause a new influenza pandemic in humans [10]. In 1999 and 2003, the reports of H9N2 AIV infections of humans in Hong Kong dramatically attracted the attention of the scientific community [11,12].

The innate immune system response is the first line of defense and limits early virus replication [13]. Cytokines are important immune mediators of innate and acquired immunity responsible for initiating, amplifying and regulating inflammation in response to pathogenic infection [14,15]. Knowledge of the innate immune recognition of AIV is crucial to understanding the viral pathogenesis in birds. Recognition of pathogen-associated molecular patterns (PAMPs) by host pattern recognition receptors (PRRs) represents a critical step in innate immune responses [16]. Proper communication between host cells and pathogen via PAMPs and PRRs initiates signal transduction pathways which in turn induces the expression of cytokines aimed at controlling pathogens [17].

Recognition of the virus through PRRs, such as retinoic-acid inducible gene I (RIG-I), melanoma differentiation-associated gene 5 (MDA-5) and toll-like receptors (TLRs) induces activation and translocation of transcription factors to nucleus, including activator protein-1 (AP-1), interferon regulatory (IRF) factor 3 (IRF-3) and nuclear factor-kappa β (NF-κβ), which collaborate to induce transcription of various cytokines such as alpha/beta interferon, leading to clearance of the infectious pathogens [18]. Understanding the mechanisms that regulate innate immune responses to AIV is of key importance to develop novel virus-based therapeutic strategies. In mammals, studies have been carried out to examine the transcription factors signaling pathways after infection with influenza virus*,* however, in chicken very little is known [19]. Although no commercial kit exists to examine chicken cellular transcription factors, previous reports have confirmed the cross-reactivity between birds and mammals in c-Jun, IRF-3 and p50 [20-24].

Lesions and viral antigen distribution are frequently observed in chicken liver infected with AIV [25-27]. Primary chicken embryo hepatocytes (CEHs) have been used for virus propagation, detection, and subsequent vaccine production [28-30]. Here we used primary CEHs which are readily used to study AIV infections because of their high susceptibility, thus are suitable to detect changes in gene expression early in the course of infection under controlled conditions. In this study, we compared viral replication, virus-induced cytokine gene expression and activation of cellular transcription factors associated with low pathogenic H5N3, H5N9, H7N2 and H9N2 viruses infection of CEH. The objective was to understand the early immune and cellular responses to broaden our understanding of the molecular mechanisms related to LPAIV infection in chicken.

## Results

### Growth kinetics of viruses on CEH

**Figure 1:**
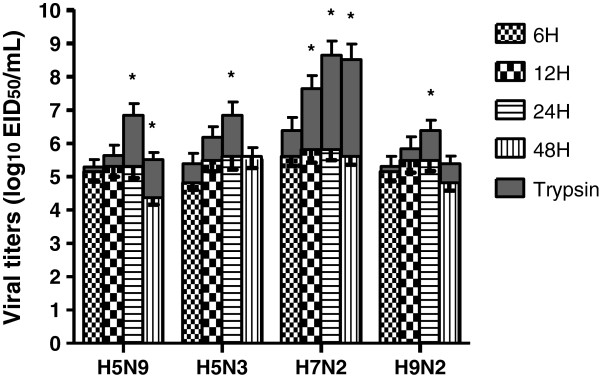
**Kinetics of CEH infection by H5N9, H5N3, H7N2 and H9N2 viruses.** Cells were infected at MOI of 1 and supplemented with1μg/ml trypsin or without trypsin in the medium. The viral titers in supernatants collected at 6, 12, 24 and 48 hpi were determined as log10EID50/ml. The patterns bars represent the viral titers achieved without the use of supplemental trypsin. The gray bar stacked on top represents the increase in the viral titers with the addition of supplemental trypsin. Error bars show standard deviation of the mean, n = 3. *Indicates the difference (P < 0.05) between the supplemented with and without trypsin group.

To investigate the replication of different virus strains, CEHs were infected with H5N9, H5N3, H7N2 and H9N2 viruses at MOI of 1 and the viral titers in the supernatants were determined as log10EID50/ml. Comparison of the growth characteristics of H5N9, H5N3, H7N2 and H9N2 viruses in CEHs with or without trypsin supplementation in the medium after infection are shown in Figure 1. CEH cannot efficient support growth of the above viruses without supplemental trypsin in the medium, presumably because CEH cannot produce trypsin-like protease. But after with1 μg/ml trypsin supplementation in the medium after infection, viral titers increasing until 24 hpi, especially H7N2 virus has a significant increase. The viral titers for H5N9, H5N3, H7N2 and H9N2 at 24 hpi were 6.8, 6.8, 8.6, and 6.4 log10 EID50 per ml, respectively.

### Pro-inflammatory IL-6/IL-1β expression following virus growth on CEH

**Figure 2:**
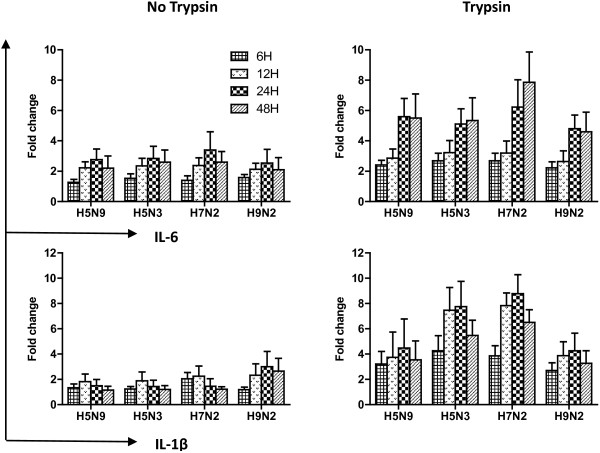
**Pro-inflammatory IL-6/IL-1β mRNA expression of CEH infection by H5N9, H5N3, H7N2 and H9N2 viruses.** Cells were infected for different time periods with LPAIV at MOI of 1 and supplemented with 1 μg/ml trypsin or without trypsin in the medium. Total RNA was isolated and quantitated using QRRT-PCR. The horizontal axis represents virus. The vertical axis represents the fold change. Error bars represent standard deviation across each condition performed in triplicate.

The influence of LPAIV on pro-inflammatory IL-6/IL-1β cytokines expression in CEH (6, 12, 24 and 48 hpi) is shown in Figure 2. The expression of IL-6 was similar in CEH with trypsin supplementation after infection at the early stage of viral infection, with a low expression level at 6 hpi and then a slight increase at 12 hpi. However, IL-6 was significantly upregulated at 24 and 48 hpi. H7N2 virus demonstrated the highest expression level of IL-6 expression at 7.9-fold increase compared to sham-infected cells at 48 hpi.

IL-1β expression was similar in CEH with trypsin supplementation with a peak at 24 hpi and decreased at 48 hpi. H7N2 also demonstrated the highest level of expression (8.8-fold) at 24 hpi. Interestingly, the IL-6 and IL-1β expression in CEH without trypsin after infection were far lower compared to expression observed with trypsin.

### Interferon-α and Myxovirus (Mx) expression following virus growth on CEH

**Figure 3:**
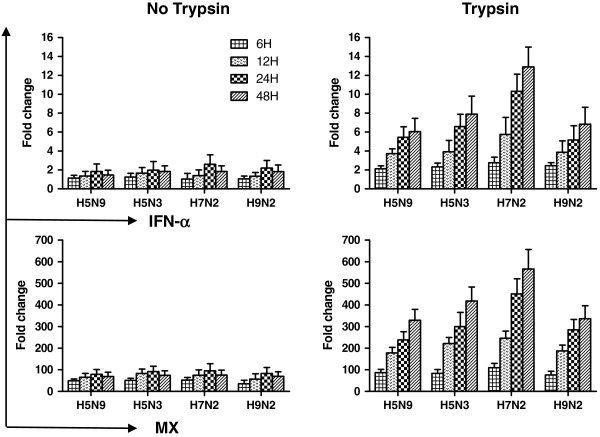
**Interferon-α/Mx mRNA expression of CEH infection by H5N9, H5N3, H7N2 and H9N2 viruses.** Cells were infected for different time periods with LPAIVs at MOI of 1 and supplemented with 1 μg/ml trypsin or without trypsin in the medium. Total RNA was isolated and quantitated using QRRT-PCR. The horizontal axis represents virus. The vertical axis represents the fold change. Error bars represent standard deviation across each condition performed in triplicate.

The induction of IFN-α and Mx expression in CEH (6, 12, 24 and 48 hpi) following LPAIV infection is shown in Figure 3. The results show that IFN-α and Mx expression pattern in the CEH with trypsin after infection are similar, with isolates inducing upregulated expression throughout the experiment. A significant increase was observed when trypsin was added compare to without trypsin. The H7N2 virus was observed to induce much higher IFN-α and Mx expression on CEH with trypsin than other viruses.

### Activation of c-Jun, p50 and IRF-3 by LPAIVs

**Figure 4:**
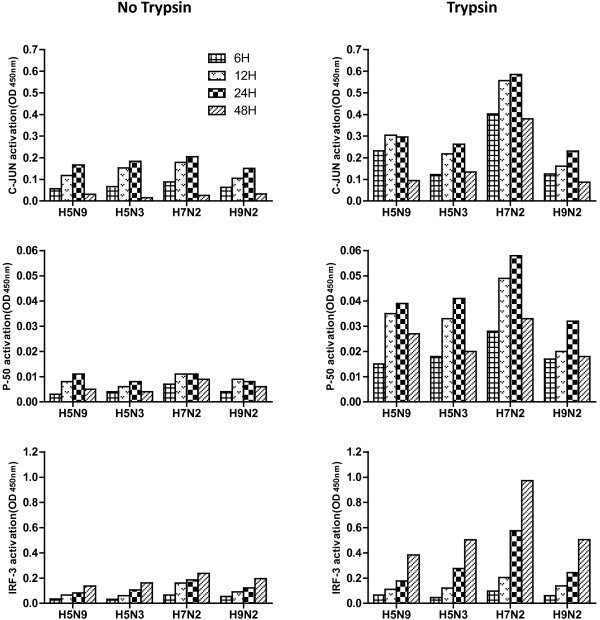
**C-Jun, p50 and IRF-3 activity in CEH in response to LPAIVs.** Cells were infected for different time periods with LPAIVs at MOI of 1 and supplemented with 1 μg/ml trypsin or without trypsin in the medium. Cell lysates (10μg/ml) were tested for binding of the activated c-Jun, IRF-3 and p50 subunits using the Trans-Am ELISA kit. The results are expressed as specific binding (absorbance measured in the presence of the mutated oligonucleotides minus that measured in the presence of the wild-type oligonucleotides) according to the manufacturer’s instructions and are compared to non-infected control.

Infection of the CEH with LPAIV induced an increase in DNA-binding transcription factors, c-Jun, p50 and IRF-3, most notably with trypsin supplementation (Figure 4). The activation patterns of c-Jun and p50 were similar in CEH with a peak at 24 hpi and decreased levels at 48 hpi. Likewise, LPAIV induced an increase in IRF-3 with all isolates upregulating expression throughout the experiment. The H7N2 virus induced much higher increase in c-Jun, p50 and IRF-3 on CEH than the other viruses.

## Discussion

In these studies we compared the effects of different LPAIV on transcription factor activation and immune response gene expression in primary CEH. For the first time we demonstrate that chicken cells increase activation of c-Jun, IRF-3 and p50 transcription factors after AIV infection, which correlated with increased cytokine expression. Transcription factor activation and immune response gene induction also correlated with virus growth.

Early after infection, viruses are capable of triggering a series of intracellular events which may be accompanied by changes in host gene expression and activation of a variety of intracellular signaling pathways that are in part exploited by the virus to ensure efficient replication [31]. In mammals, influenza virus infection and replication result in the production of multiple proinflammatory, chemotactic and antiviral cytokines [32,33]. Cytokine gene expression are associated with the activation of a series of signal transducing molecules, such as AP-1 [34], STAT [35] and IRF [36], in influenza virus infected cells. However, our understanding of which transcription factors are involved in the cytokine gene expression mounted by the chicken cells to AIV is unknown.

When CEHs were infected with LPAIVs in the presence of trypsin, viral titers were increased, especially the H7N2 virus. This result suggests that LPAIV cannot replicate very well in CEH without exogenous trypsin. This is consistent with previous reports that report a need for trypsin-like proteases to cleave the hemagglutinin protein into the HA1 and HA2 subunits, which is required for the virus to be infectious [37,38].

Infection with HPAI in birds and mammals is associated with severe pathology and increased mortality. One possible hypothesis for the cause of death in mammals due to HPAI infection is the acute induction of high levels of inflammatory cytokines, a so called cytokine storm [39-41]. The results of our study also demonstrate increased expression of pro-inflammation cytokines, IL-6, IL-1β and IFN-α, following LPAIV infection *in vitro*. These results correlate with previous studies which have also demonstrated increased pro-inflammatory cytokines expression following LPAIV infection [42,43].

Interferon regulatory factors are a family of DNA-binding proteins involved in mediating the cellular immune response following viral infection. In mammals influenza virus infection induces activation of IRF-3 signaling [44-46], however its role in chicken immunity remains in question. It has been reported that chicken IRF-3 is activated by type I and type II IFN and its binding specificity has been demonstrated [47,48]. More recently, Liniger reported that chicken IRF-3 was required for virus-mediated type I IFN induction in DF-1 cells [49]. In our study, we also demonstrate increased IRF-3 production after infection with all isolates of LPAIV tested.

The transcription factor NF-κβ is known as a major regulator of the inflammatory response [50,51]; however, its role in avian influenza virus replication and virus-induced immune response is ill-defined. In recent studies, NF-κB subunit p50 knockout cells demonstrated that activation of p50 is obsolete for production of interferon stimulated genes (ISG) upon virus infection [52] and that p50 is the predominant negative regulator of ISGs in the context of influenza virus infection [53]. In our studies, we observed that infection with LPAIV induced an increase in p50 DNA binding after infection. Furthermore, the increased activation of p50 correlated with a resulting increase of proinflammatory cytokines. A previous study has reported that treatment of chicken heterophils with either flagellin or lipopolysaccharide induced a significant increase in DNA binding by the NF-κB family member p50 [54]. These results demonstrate the significant role of p50 activation in inducing the expression of pro-inflammatory cytokines in chicken following infection.

In mammals, influenza virus infection induces transcription factor c-Jun and AP-1 activation and signaling, and helps to generate many of the biological effects of IFN production [34,44-46,55-59]. However in gallinaceous birds very little was known about the signaling pathways after infection with AIV. We observed an increase in c-Jun activation after infection of CEH with LPAIV. The increased transcription factor activation correlated with increased proinflammatory response, these sequential processes suggest that c-Jun and IRF-3 likely mediate the induction of IL-6, IL-1β and IFN-α gene expression. Similar timing of transcription factor activation and cytokine gene expression was found in mammalian cells [34,45]. To our knowledge these findings confirm for the first time, that similar to mammal cells, infection of chicken cells with LPAIV increases protein expression of host DNA-binding transcription factors.

## Conclusion

Our results demonstrate CEH can efficiently support growth of the LPAIV with trypsin supplementation in the medium after infection, especially the H7N2 virus. After infection all immune response genes tested were upregulated and the transcription factors, c-Jun, IRF-3 and p50, were also increased compared to sham-infected controls. These data will broaden our understanding the avian immune response to infection with LPAIV and has implications for strategies that target the innate immune system for improving resistance to avian influenza.

## Materials and methods

### Cells isolation and culture

Primary CEHs were isolated from 14-day-old white leghorn chicken embryos of Specific Pathogen Free (SPF) eggs as previously described [60] and were cultured at 37°C and 5% CO2 in Dulbecco’s modified Eagle’s medium (DMEM) supplemented with 10% fetal bovine serum (Invitrogen, CA,USA), 2 mM glutamine and antibiotics (final concentration: penicillin, 100 U/ml; streptomycin, 100 μg/ml). Cell viability was assessed by the Trypan Blue exclusion test and was not less than 90% for each preparation. Contamination by non-hepatocyte cells was minimal by microscopic examination. The cells were counted and suspensions were diluted to 2 × 106 viable (dye-excluding) cells per ml. The cells were distributed into 60 × 15 mm culture dishes (Becton Dickinson, NJ, USA) for virus growth kinetics and RNA extraction or nuclear extraction, respectively. For each experiment, cells were prepared on the same day and under the same conditions.

### Virus and cell culture infection

A/turkey/Wisconsin/68 (H5N9), A/chicken/Texas/167280-4/02 (H5N3), A/turkey/Virginia/158512/02 (H7N2) and A/chicken/NJ/12220/97 (H9N2) LPAI viruses were propagated in allantoic cavities of 9–11 day of embryonation SPF chicken eggs. Viral titers were determined as previously described [61]. All experiments using infectious virus were conducted in bio-safety level 2 (BSL-2) facilities at the Southeast Poultry Research Laboratory (SEPRL), Agricultural Research Service, United States Department of Agriculture (USDA).

### Virus growth curves

The growth curves of the viruses were determined by virus titration of cell culture supernatants at different time points after infection of primary CEH. Briefly, cells were infected with each virus at a multiplicity of infection (MOI) of one in DMEM, negative control cells were set up identically but without the addition of virus. Culture dishes were gently rocked every 15 min for 1 h at 37°C, non-adsorbed viruses were removed and the cells were washed with sterile phosphate buffered saline (PBS). DMEM supplemented with 2% FBS or 1 μg/mL TPCK trypsin (Sigma–Aldrich, St. Louis, MO) for CEHs were added per dish and the dishes were incubated at 37°C and 5% CO2. At 6, 12, 24, and 48 hours post-infection (hpi), supernatants were collected and stored at -80°C until used for titrations in SPF embryos. Virus titers were determined using the method of Reed and Muench and expressed as 50% egg infectious dose (EID50) [62].

### Isolation of RNA and quantification of cytokine gene expression

Total RNA was isolated from infected and control cells at each time point using the RNeasy mini kit (Qiagen) in accordance with the manufacturer’s instructions, and treated with RNase-free DNase I (Invitrogen) to remove genomic DNA. All RNA samples were checked by using a Nanodrop spectrophotometer (Nanodrop Technologies, Wilmington, DE). Relative cytokine mRNA expression in the abovementioned cells were examined by quantitative RT-PCR. Interleukin (IL)-1β, IL-6, IFN-α, and Mx expression were determined as previously described [15,63]. Briefly, quantitative PCR was performed for each sample in triplicate in a total volume of 25 μl, consisting of 12.5 μl iQ SYBR Green supermix (Bio-Rad Laboratories, CA, USA) with 1 μl of each primer at concentration of 10 pmol/μl, 0.5 μl iScript reverse transcriptase, 5 μl RNase/DNase-free water, and 5 μl diluted RNA. PCR conditions were the same for each targeted gene and are as follows: 10 min at 50°C, 95°C for 5 min, followed by 45 cycles of 95°C for 10 s and 58°C for 30 s. For each reaction, melting curves were analyzed to determine the specificity of each gene. Primers were derived from previously published sequence and synthesized by Integrated DNA Technologies [64]. Sequences are as follows (forward, reverse): IL-6 (5’-GCGAGAACAGCATGGAGATG-3’, 5’-CTGTTCGCCTTTCAGACCTAC-3’); IL-1β (5’-ACATGTCGTGTGTGATGA-3’, 5’-GCTTCATCTTCTACCGCCTG-3’); IFN-α (5’-GACAGCCAACGCCAAAGC-3’, 5’-AATGCTTGAGCAGCAGCGAC-3’); Mx (5’-CAGGACATCAACGACAATCT-3’, 5’-TTGCCAGATGAGGGATAGTA-3’) and 28S (5’-GGCGAAGCCAGAGGAAACT-3’, 5’-GACGACCGATTTGCACGTC-3’). RNA from individual cell samples was normalized using 28S house-keeping gene. For each gene, amplification was verified using four 10-fold serial dilutions of standard spleen cell RNA in the same PCR run. Expression was determined by the standard curve method [65]. Data are expressed as fold change in cytokine messenger RNA (mRNA) levels in infected groups compared with those from uninfected groups.

### c-Jun, IRF-3 and p50 activation analysis

The ELISA-based Trans-Am transcription factor kits (Active Motif, Carlsbad, CA, USA) were used to detect and quantify c-Jun, IRF-3 and p50 activation as previously described [54]. The 96-well plates are contain immobilized oligonucleotides containing transcription response elements for c-Jun (5′-TGAGTCA-3′), NF-κβ p50 consensus binding site (5′-GGGACTTTCC-3′) or IRF consensus binding site (5′-GAAACTGAAACT-3′). The active forms of the subunits for c-Jun or p50 or IRF-3 in nuclear extracts can be detected using specific antibodies for epitopes that are accessible only when the nuclear factors are activated and bound to their target DNA. Preparation of nuclear extract was done according to the manufacturer’s instructions. The specificity of the assays was checked by measuring the ability of soluble wild type or mutated c-Jun, p50 and IRF-3 oligonucleotides to inhibit binding. The results are expressed as specific binding (absorbance measured in the presence of the mutated oligonucleotides minus that measured in the presence of the wild-type oligonucleotides) according to the manufacturer’s instructions.

### Statistical analyses

Data are expressed as the mean ± standard deviation. Statistical differences were analyzed with Tukey one-way ANOVA using Prism 5 (GraphPad Co., San Diego, CA).

## Competing interests

The authors declare that they have no competing interests.

## Authors' contributions

HJ and DRK carried out virus growth on cell culture as well as RRT-PCR for avian cytokines. HJ performed transcription factor analysis. KY participated in study design and coordination. HJ and DRK wrote the manuscript. All authors read and approved the final manuscript.
